# Non-Catalytic Roles of the Topoisomerase IIα C-Terminal Domain

**DOI:** 10.3390/ijms18112438

**Published:** 2017-11-17

**Authors:** Duncan J. Clarke, Yoshiaki Azuma

**Affiliations:** 1 Department of Genetics, Cell Biology & Development, University of Minnesota, 420 Washington Ave SE, Minneapolis, MN 55455, USA; 2 Department of Molecular Biosciences, University of Kansas, Lawrence, KS 66045, USA

**Keywords:** Topoisomerase II, SUMO, C-terminal domain, mitosis, Aurora B, Haspin, Claspin, metaphase checkpoint

## Abstract

DNA Topoisomerase IIα (Topo IIα) is a ubiquitous enzyme in eukaryotes that performs the strand passage reaction where a double helix of DNA is passed through a second double helix. This unique reaction is critical for numerous cellular processes. However, the enzyme also possesses a C-terminal domain (CTD) that is largely dispensable for the strand passage reaction but is nevertheless important for the fidelity of cell division. Recent studies have expanded our understanding of the roles of the Topo IIα CTD, in particular in mitotic mechanisms where the CTD is modified by Small Ubiquitin-like Modifier (SUMO), which in turn provides binding sites for key regulators of mitosis.

## 1. Introduction

**Figure 1:**
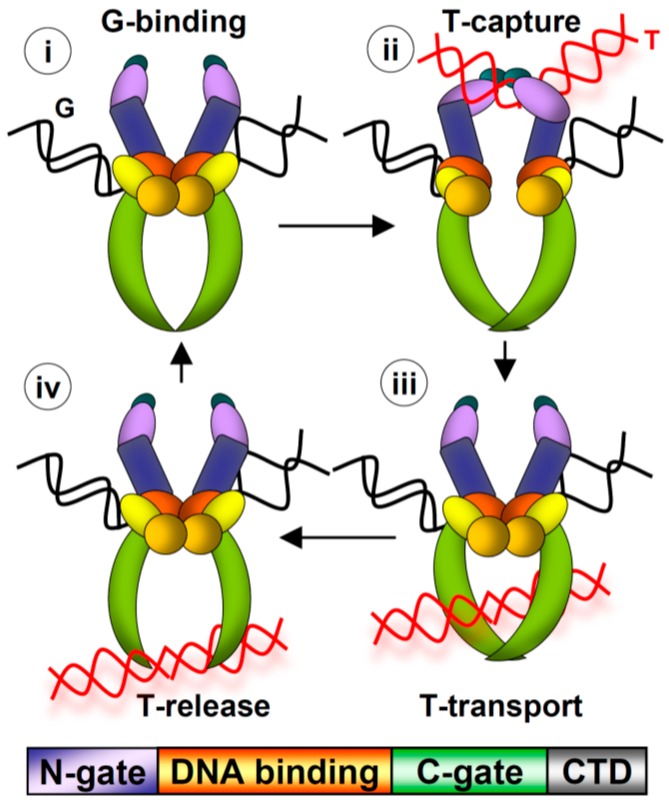
Top: Strand Passage Reaction. (**i**) G-segment DNA binds at catalytic core (orange/yellow/red), (**ii**) T-segment DNA captured by N-gate (purple/blue), G-segment cleavage, (**iii**) T-segment transport, G-segment ligation, (**iv**) T-segment release from C-gate (green). Bottom: Domain structure (colors match domains above). C-terminal domain (CTD; grey) has not been crystalized.

In this review, we summarize recent studies that have revealed insight into the non-catalytic functions of the Topo IIα CTD. Topo IIα and Topoisomerase IIβ (Topo IIβ) are the two vertebrate Type II topoisomerases. These proteins are unique in that they are the only enzymes that perform the strand passage reaction, in which a double helix of DNA is broken, a second double helix is transported through the break, and then the first helix is re-ligated (Figure 1). Based on the fidelity and rapid kinetics of this reaction, Type II topoisomerases can resolve intertwined DNA helices without generating deleterious DNA breaks. Topo IIα is the subject of study here. The strand passage reaction of Topo IIα is essential for mitotic processes, including chromosome condensation and segregation. Intriguingly, the mitotic role of Topo IIα cannot be substituted by Topo IIβ although both have comparable strand passage reaction activity [1]. Moreover, swapping their distinct CTDs reversed the roles of Topo IIα and Topo IIβ in mitosis, suggesting that the CTD carries essential functions for the mitotic role of Topo IIα [2]. (The roles of Topo IIβ, which are largely non-mitotic, will not be discussed). The CTD of Topo IIα has been shown to be dispensable for the strand passage reaction [3,4], but nevertheless evidence has revealed that the CTD is crucial for the fidelity of chromosome segregation in yeast, human cells, and Xenopus egg extract (XEE) [5,6,7]. This led to the hypothesis that the CTD has fundamental conserved functions independent of the strand passage reaction that are required for accurate genome transmission. The data pertaining to these mitotic functions will be discussed.

## 2. Evidence That the CTD Mediates Functional Interactions with Chromatin

A longstanding gap in our knowledge about Topo IIα is the mechanism of recruitment to and interaction with chromatin. Initial discoveries revealed that Topo IIα is a major component of mitotic chromosomes [8,9,10]. Beyond this simple finding, however, there remains much to discover about how and where Topo IIα associates with chromatin [11]. Biochemical purifications using mitotic chromatin found Topo IIα within a chromosomal scaffold fraction, which is a structure that remains on the chromosomes after high salt extraction [8]. The condensin complex was also found within this structure. Studies using the XEE cell-free assay system showed that the immuno-depletion of either Topo IIα or condensin proteins led to defects in chromosome condensation [12,13]. This mitotic condensation function has since been found to be conserved in many organisms. Since Topo IIα and condensin both participate in mitotic chromosome condensation, this implies a functional relevance of the localization within the chromosome scaffold. Moreover, it suggests the potential role of Topo IIα and condensin in the organization of mitotic chromosomes. For example, one hypothesis supposes that Topo IIα is a structural component of chromosomes and that loops of chromatin may be organized by a scaffold composed of Topo IIα and condensin.

As well as collaborating with condensin, Topo IIα has roles in DNA replication and chromosome segregation [14,15]. This indicates that Topo IIα cannot be restricted to the chromosome scaffold, and, indeed, cytological analyses largely corroborate this idea [11]. When mitotic chromosomes were immuno-stained with anti-Topo IIα (or anti-condensin) antibodies, Topo IIα was generally seen to be concentrated within a central region of the chromosome arms (now termed the chromosome core or axis) [7,9,10,16,17,18]. However, in live cells, Topo IIα fused to fluorescent proteins can be observed across the entire width of chromosomes [19]. This was also the case when rhodamine-labeled Topo II was observed in Drosophila embryos [20]. In some cases, such as the large chromosomes of Indian muntjac cells, Topo IIα can appear more concentrated within the central chromosome core than the whole chromosome arm’s width, even in live cells [7]. However, one particular study shed light on these somewhat variable results, revealing that the core localization pattern is largely seen only after the hypotonic treatment of cells or after fixation [19]. Perhaps, altogether, these studies have revealed that the association of Topo IIα with chromatin is not regulated uniformly and that mechanisms of targeting and retention will differ at various genomic sites. Although the mechanisms of targeting Topo IIα to the chromatid cores and to chromatin in general are not understood, recent evidence implicates the CTD in the molecular interface between Topo IIα and chromatin.

Firstly, purified recombinant human Topo IIα CTD (residues 1321–1531) can bind to plasmid DNA and short DNA oligonucleotides in vitro [7]. Interestingly, the Topo IIβ CTD (residues 1359–1621) has a much lower affinity for DNA in the same assays, indicating that the affinity of human Topo IIα CTD for DNA could be part of the mechanism that sets these two domains apart. This is consistent with fluorescence anisotropy data measuring the affinity of Topo IIα and Topo IIβ, as well as versions lacking the CTDs, for DNA [21]. These studies indicate that the Topo IIβ CTD acts as a negative regulator of the enzyme in that the presence of the CTD reduces the affinity for DNA. Further, activity assays have suggested that the Topo IIβ CTD inhibits, while the Topo IIα CTD stimulates, strand passage [22]. The data suggest that DNA affinity could be part of the enhanced ability of human Topo IIα CTD to interact with mitotic chromosomes and perform chromosomal functions in mitosis. It is not known if the Topo IIα CTD preferentially binds to DNA at the chromatid cores.

**Figure 2:**
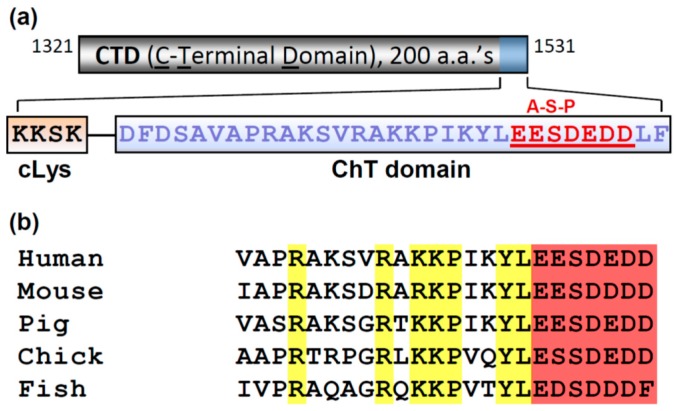
Components of the human Topo IIα CTD: (**a**) 200 a.a. CTD (grey box); the last 31 residues define the Chromatin Tether (ChT) domain (**shaded blue**). ChT domain (blue box) with Acid-Patch nested serine motif (A-S-P; red) and the upstream cluster of Lysine (cLys) residues (orange box); (**b**) conserved ChT residues (**yellow**) of Topo II adjacent to the A-S-P motif (**red**).

Secondly, purified recombinant human Topo IIα CTD (residues 1321–1531) can precipitate histone H3 from HeLa cell extracts, and this ability is dependent on features at the extreme C-terminus of the CTD (Figure 2), which has been termed the Chromatin Tether (ChT; the last 30 residues) [7]. The deletion of as few as the last 11 residues abolished the ability of the CTD to precipitate H3 [7]. These residues span a highly conserved acidic patch with a nested serine residue which is a known phosphorylation site [23]. Whether this acidic region directly interacts with basic H3 residues has not been tested, but it is intriguing to consider if the phosphorylation site regulates such an interaction. In combination with the evidence in favor of direct interaction with DNA, we may speculate that the CTD interacts with nucleosomes. This seems to be at odds with evidence that Topo IIα preferentially interacts with genomic loci that have low nucleosome occupancy (discussed in ref. [7]). However, perhaps the CTD contributes to a specialized function of Topo IIα by stimulating the interaction with nucleosomes in circumstances where histone density is not low, for example as may occur during chromosome condensation in mitosis.

Adding further complexity is the fact that mitotic chromosomes have two distinct populations of Topo IIα in terms of their resistance to high salt. This has been demonstrated in both XEE and mammalian cells [5,8,10]. Which of these populations of Topo IIα most directly contributes to chromosome condensation is not clearly defined, though an interesting finding is that the salt-stable fraction increases in mitosis [24]. Importantly, the inhibition of modification by SUMO (SUMOylation) can increase the salt-resistant population of Topo IIα in mitotic XEE assays [5]. This may be because the increased salt-resistant population of Topo IIα represents catalytically active Topo IIα on mitotic chromosomes, and Topo IIα activity might be higher in the complete absence of SUMOylation due to a lack of SUMOylation at an inhibitory Lysine 660 residue within the DNA-binding region of Topo IIα [25]. Other studies have provided evidence that Topo IIα SUMOylation within the CTD by specific E3 ligases can stimulate binding to mitotic chromosomes [26,27]. In mice, the lack of E3 SUMO ligase RanBP2 compromised the targeting of Topo IIα to inner centromeres in mitosis [27]. In human cells, the depletion of E3 SUMO ligase PIASγ reduced the accumulation of Topo IIα on mitotic chromosomes [26]. One explanation of these findings is the potential effect of chromatin factors that bind with increased affinity when the CTD is SUMOylated. Consistent with the consequence of the SUMOylation of several SUMOylated proteins [28], the SUMOylation of the Topo IIα CTD promotes novel binding to cellular proteins that have a SUMO interacting motif (SIM). Therefore, it is possible that SUMOylation-dependent binding proteins residing at the Topo IIα CTD influence CTD binding to nucleosomes in mitotic chromatin.

The in vitro biochemical interactions of the human Topo IIα CTD with DNA and histones correlates with functional readouts of Topo IIα function in mitosis. Firstly, the dynamics of Topo IIα in mitosis are dictated in part by the CTD. Fluorescence recovery after photo-bleaching (FRAP) studies have revealed that Topo IIα is highly mobile on mitotic chromosomes, and moreover that the entire cellular population of Topo IIα is mobile [7,17,19]. There is no understanding as to why Topo IIα should be highly dynamic, nor is there strong evidence that this high mobility is biologically important. Nevertheless, removing the ChT residues from the extreme end of the CTD both abolishes the in vitro interaction with H3 and leads to even higher mobility of Topo IIα in mitosis [7]. In human cells, this was established experimentally by expressing an exogenous copy of either the Topo IIα ΔChT mutant fused to mCherry or a wild-type Topo IIα–mCherry fusion. The half-life of the Topo IIα ΔChT mutant protein on metaphase chromosomes was reduced by about 40%. This indicates that the Topo IIα ΔChT mutant has a lower affinity for chromatin than wild-type Topo IIα–mCherry (since bleached Topo IIα ΔChT–mCherry was replaced more quickly by unbleached protein from the soluble pool in the FRAP studies). Even in the absence of functional data, this correlation suggests that H3 (and perhaps DNA) binding contribute to the dynamics of Topo IIα in vivo. This would indicate that the bulk of Topo IIα relies in part on H3 interaction for tethering it to chromatin in mitosis. To test the possible functional relevance of Topo IIα mobility, the endogenous Topo IIα was depleted from cells and at the same time either Topo IIα ΔChT–mCherry or wild-type Topo IIα–mCherry was expressed at close to endogenous levels. When mitotic chromosomes were examined in the Topo IIα ΔChT mutant they were found to be abnormal, with a lack of sister chromatid resolution and poor longitudinal condensation in metaphase [7]. These are hallmark phenotypes of a loss of Topo IIα function [29,30], providing circumstantial evidence that the residence time of Topo IIα on chromatin or the interaction with H3 in particular is biologically important. It is established that the CTD of Topo IIα is dispensable for the strand passage reaction, though the data indicate that the CTD provides substrate specificity wherein the preferred DNA conformation is positively supercoiled DNA [4]. If the contribution of the CTD were examined in the context of chromatin, rather than naked DNA, then it is possible that such studies would reveal additional roles due to interaction with H3. One possibility is that the enzyme requires its normal residence time on chromatin to productively process catenated DNA to allow condensation and sister resolution. The 40% reduction in half-life seen in the Topo IIα ΔChT mutant could render decatenation non-productive. Alternatively, H3 interaction could have a mechanistic consequence beyond simply dictating the residence time of Topo IIα on chromatin. In vitro, the Topo IIα CTD binds specifically to the histone H3 N-terminal tail, and phosphorylation of the H3 tail has been long known to be important for the process of chromosome condensation [31,32,33]. This could mechanistically link H3 tail phosphorylation with the role of Topo IIα in condensation. Since in XEE and yeast cells, the Topo IIα CTD has an important role in recruiting the kinase that phosphorylates the H3 tail (Aurora B) to chromatin [34,35], it is plausible that Topo IIα-mediated H3 phosphorylation contributes to chromosome condensation [36].

## 3. The Topo IIα CTD Serves as a Scaffold to Recruit Mitotic Regulators to Centromeres

Specifically from early mitosis until the onset of anaphase, Topo IIα is enriched at the centromere regions of chromosomes [19]. Indeed, Topo IIα has been shown to be actively engaged in decatenation reactions at centromeres in G2 and in mitosis, and cleavage sites have been mapped to centromeric α-satellite DNA sequences [37]. Since the α-satellite sequences of DNA are unique centromeric features, it is possible that this enriched localization is based on a preference for binding to centromeric DNA. One argument against this is the finding that Topo IIα associates equally with native centromeres and active neocentromeres that do not contain α-satellite DNA [38]. In fact, the same study showed that active Topo IIα is not present at the inactive centromere in dicentric chromosomes. Therefore, Topo IIα localization appears to be based on protein features of active centromeres. Whether this includes centromeric histones has not been tested, but it is consistent with evidence that centromeric sites of DNA cleavage by Topo IIα are likely to be determined epigenetically [39]. Recent evidence has revealed centromeric Topo IIα functions that rely on centromeric histones and involve SUMOylation of the Topo IIα CTD.

Topo IIα CTD SUMOylation is conserved from yeast to vertebrates [5,6,27,40]. Mice in which Topo IIα CTD SUMOylation is compromised have increased frequencies of mitotic chromosome segregation errors, aneuploidy, and tumor incidence [27]. In XEE assays, Topo IIα SUMOylation is spatiotemporally restricted to the early stages of mitosis and to centromeres [41,42]. The potential role of this modification in mitosis became apparent after the identification of binding proteins that specifically interact with the SUMOylated Topo IIα CTD [35,40]. Two proteins were identified, Claspin and Haspin, both of which regulate the key mitotic kinase Aurora B. Moreover, Claspin and Haspin were specifically recruited to centromeres in mitosis by the SUMOylated Topo IIα CTD. This is an important link because Aurora B localizes, and performs key functions, predominantly at centromeres in mitosis. Claspin is known to interact with Chk1, a kinase that has a role in the activation of Aurora B in human cells via phosphorylation of residue S331 [43]. Haspin kinase, on the other hand, promotes the efficient recruitment of Aurora B to centromeres through the phosphorylation of histone H3 residue T3 (H3T3-Phos) [34,44,45,46]. The H3T3-Phos modification is largely confined to centromeres in mitosis, and it is recognized by Survivin in the Chromosome Passenger Complex (CPC), which also contains Aurora B [46]. In this manner, Topo IIα CTD SUMOylation is directly involved in the regulation of Aurora B activity at mitotic centromeres.

## 4. Functions of the CTD at Yeast Centromeres

The budding yeast Saccharomyces cerevisiae has a single Type II topoisomerase, Top2. The deletion of the gene encoding Top2 can be complemented by either Topo IIα or Topo IIβ from human cells [47]. Therefore, the enzymes are sufficiently conserved to carry out the essential functions of a Type II topoisomerase in yeast cells. As described above, in XEE Topo IIα concentrates at centromeres in mitosis and the evidence indicates that the SUMOylated CTD has functions at centromeres that are crucial for chromosome segregation. Similarly, the defined biochemical mechanism of Top2 function at yeast centromeres seems to rely on a conserved CTD SUMOylation-dependent scaffold for the recruitment of factors that orchestrate regulatory events in mitosis [34]. In yeast strains with the penetrant temperature-sensitive *top2-4* allele, overall kinetochore structure and function appears to be normal. The evidence for this is that outer kinetochore proteins localize normally to kinetochores in *top2-4* cells. Kinetochores are built on centromeres in a hierarchical manner in several layers. Thus, the underlying architecture on which the kinetochore is built is presumably intact in *top2-4* cells. This evidence agrees with the finding that treating mammalian cells with a concentration of the Topo IIα inhibitor ICRF-193, which can fully block sister chromatid separation in anaphase, nevertheless does not disrupt kinetochore structure as judged by electron microscopy [48], and evidence from Drosophila S2 cells that Topo II is dispensable for kinetochore structure in mitosis [49]. Indeed, it has proven difficult to assign any centromeric functions to Topo IIα despite its enrichment and demonstrated catalytic activity at centromeres in mitosis [50]. Contrasting with these data, however, yeast *top2-4* cells were found to be strongly defective in recruiting the yeast Aurora B ortholog (Ipl1) to the inner centromere during mitosis [34]. Interestingly, there was no defect in interphase cells, indicating firstly that the mechanism of Ipl1 recruitment to the inner centromere differs between interphase and mitosis, and secondly that the biological importance of Top2 in Ipl1 recruitment to inner centromeres is mitosis-specific. Further dissection revealed that the CTD is required for mitosis-specific Ipl1 recruitment. In line with the evidence in XEE, conserved CTD SUMOylation sites were found to be essential for Ipl1 recruitment in mitosis. Like in XEE, the evidence indicated that the mechanism of Ipl1 recruitment in yeast mitosis relies on the SUMOylated Top2 CTD serving as a scaffold for orthologs of the Haspin kinase (Alk1 and Alk2) to phosphorylate histone H3 on threonine 3 (H3T3-Phos). In turn, H3T3-Phos provides a binding site for the CPC of which Ipl1 is a component. The mutation of Alk1 and Alk2 abolished Ipl1 recruitment, as did mutation of the H3T3 residue to alanine. Strikingly, the expression of an H3 phospho-mimetic mutant (H3T3E) was able to partly rescue the defect in mitotic Ipl1 recruitment seen in both the *top2ΔCTD* and *alk1Δalk2Δ* mutants. Therefore, the requirements for the Top2 CTD and the Haspin kinases are bypassed by mimetic phosphorylation of the T3 residue. The simplest way to explain these results is that Top2 and Haspin act upstream of H3T3 phosphorylation, specifically to recruit Ipl1 to the inner centromere in mitosis. Sgo1 is also needed for Ipl1 recruitment, but it has not been fully resolved if Sgo1 works in the same pathway as Top2, Haspin, and H3T3. In other species, Sgo1 seems to recruit Aurora B by bridging an interaction between the CPC and a phosphorylated species of H2A at centromeres. Since the localization of Sgo1 to centromeres was normal in the *top2-4* mutant, Top2 does not recruit Sgo1. It remains possible that Sgo1 is required for Top2 recruitment. Otherwise, it may be that interactions with both H3T3-Phos and H2A-Phos are needed for Ipl1 to bind securely to centromeres, and in this scenario Top2 and Sgo1 would act in parallel.

## 5. Evidence That the CTD of Budding Yeast Top2 Functions in Checkpoint Signaling

In mammalian cells, Topo IIα inhibitors that catalytically inhibit the strand passage reaction activate a metaphase checkpoint [51,52,53]. The transient metaphase delay induced by this checkpoint is Mad2- and BubR1-dependent, though interestingly Mad2 is not recruited to the kinetochores [51,53]. In budding yeast, there is a corresponding mitotic checkpoint response that becomes activated by mutant Top2 proteins that mimic the effects of the chemical inhibitors [54,55]. The best characterized of these mutant enzymes are deficient in ATP hydrolysis [55]. Yeast cells expressing these mutant alleles of Top2 delay the onset of anaphase, arresting temporarily in metaphase. The metaphase arrest is observed even in a hypomorphic mutant, *top2-B44*, where ATP hydrolysis does occur but more slowly than with a wild-type enzyme. The outcome in this case is that the enzyme completes strand passage reactions successfully, but at a reduced rate. Therefore, decatenation takes longer than in wild-type cells. The significance of this observation may be that cells are monitoring the Top2-B44 enzyme directly to ascertain if there is sufficient Top2 activity to allow sister chromatids to separate. If this were the case, then the metaphase delay would be expected to rescue *top2-B44* cells from chromosome non-disjunction and lethality. This agrees with the fact that *top2-B44* cells are viable. Moreover, abolishing the temporary metaphase arrest in *top2-B44* cells, which can be accomplished by deleting the *MAD2* checkpoint gene, results in chromosome non-disjunction and rapid lethality. This checkpoint, therefore, seems to allow anaphase to proceed only once decatenation activity is sufficient for accurate chromosome segregation. Significantly, the CTD is required for activation of this checkpoint [55]. That is, when the CTD is deleted from the Top2-B44 enzyme, the checkpoint response cannot be launched and the cells rapidly become inviable even though the downstream effectors of the checkpoint (Mad2, etc.) are intact.

One explanation of these findings could have been that catalytic *top2* mutants, such as *top2-B44*, affect chromatin in a manner that is detrimental to chromosome biorientation, thus activating the spindle assembly checkpoint (SAC) as a result of chromosome misorientation. However, several observations have ruled out this possibility, even though the checkpoint response in the *top2* mutants requires several SAC proteins, including Mad2. When the SAC detects a biorientation defect, Mad2 activation must occur at the kinetochore of that chromosome. An elaborate mechanism recruits Mad1–Mad2 complexes to the kinetochore, from where the complex is stored at Nuclear Pore Complexes (NPC) when the checkpoint is off [56,57,58]. Thus, the SAC has a strict requirement wherein kinetochores are essential for the generation of the checkpoint signal (i.e., for Mad2 activation) [59,60]. However, the checkpoint response in *top2* mutants does not require kinetochores [55]. Further, Mad2 is not recruited to kinetochores when the checkpoint is active in *top2* mutants: Mad2 remains at the NPCs [55]. Logically, Mad2 is activated at NPCs in *top2* mutants, not at kinetochores. A landmark paper demonstrated that Mad2 can be activated at NPCs in human cells prior to mitosis [61]. This indicates that Mad2 activation at NPCs may occur through a conserved mechanism.

**Figure 3:**
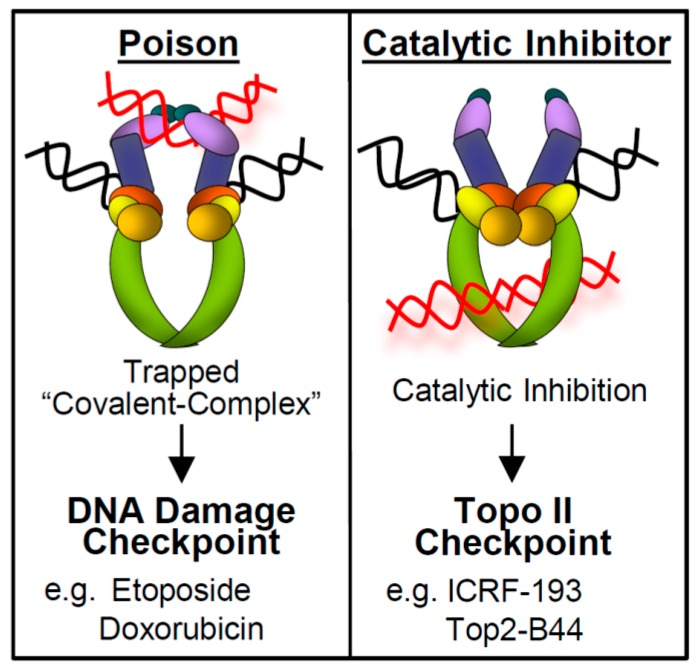
Comparison of Topoisomerase II (Topo II) poisoning (**left**) versus catalytic inhibition (**right**). DNA color scheme: Black helix (G-segment DNA); Red helix (T-segment DNA). Topo II domains: N-gate (purple/blue); DNA-binding catalytic core (orange/yellow/red); C-gate (green).

How might cells monitor Top2 to ensure that decatenation is complete before anaphase? As above, the defect in Top2-B44 is a slow rate of ATP hydrolysis. This translates to about a six-fold decrease in the rate of decatenation, but nevertheless the strand passage reaction completes and is not arrested. Can cells detect slow Top2 strand passage reactions? Or are remaining catenations themselves detected? Several reports addressed the latter possibility using degron *TOP2* alleles, where the Top2 proteins could be eliminated in the G1 phase of the cell cycle, allowing for progression through the S-phase in the absence of Top2 [55,62]. Here, cells reach mitosis with hyper-catenated DNA because no catenations can be resolved while the genome is being replicated. Nevertheless, in this scenario, the metaphase checkpoint was not activated and cells proceeded into anaphase without delay. Therefore, yeast cells are unable to detect catenations in the absence of Top2. Similarly, studies using mammalian cells and avian DT40 cells depleted of Topo II have provided evidence that a G2 decatenation checkpoint cannot detect catenations in the absence of catalytically inhibited Topo II protein [63,64]. This indicates that cells may detect the slow strand passage reactions directly. It is likely to be significant that most of the yeast *top2* mutants that cause checkpoint activation are defective in ATP hydrolysis. The lack of ATP hydrolysis is thought to slow the transit of the enzyme through quite large conformational changes that aid in moving an intact DNA helix through the central cavity of the enzyme. It could be that cells detect Top2 delayed at a particular conformation as it goes through a slow strand passage reaction. Regardless of how cells detect slow strand passage reactions, this checkpoint mechanism contrasts starkly with the activation of the DNA damage checkpoint that can occur when a Topo IIα enzyme becomes poisoned [65]. In this regard, it is important to explain that there are two contrasting modes of Topo IIα inhibition (Figure 3). As described above, mutations (or chemical inhibitors) that affect ATP hydrolysis result in slow strand passage. These are termed catalytic inhibitors. Very different mutations or chemical inhibitors can cause Topo IIα to be poisoned, where the Topo IIα enzyme becomes locked in a covalent complex with DNA. In these covalent complexes, Topo IIα has cut one DNA double helix and both active site tyrosines of the dimeric enzyme have become covalently bonded to the broken DNA ends. Since covalent complexes can degenerate (or be processed) to become recognized as DNA breaks, Topo IIα poisons activate the DNA damage checkpoint. Importantly, the catalytic inhibitors do not trap Topo IIα in covalent complexes, do not cause DNA breakage, and do not activate the DNA damage checkpoint [54,55]. Catalytic inhibitors can in fact compete biochemically with Topo IIα poisons. That is, a catalytic inhibitor will prevent covalent complex formation and DNA breakage induced by a poison [65]. Based on the different biochemical modes of action of these two classes of Topo IIα inhibitor, it is not surprising that distinct checkpoint mechanisms are employed to detect perturbed strand passage. Of course, cells must deal with DNA breakage versus incomplete decatenation very differently.

The major unknown here is how slow strand passage is sensed molecularly via the CTD of Top2 to generate a checkpoint signal that activates Mad2 at NPCs. The CTD is dispensable for the strand passage reaction and yeast cells lacking the CTD are viable but have an elevated frequency of chromosome loss. This indicates that the CTD has important functions for chromosome stability, which include the mounting of the checkpoint response when Top2 activity is not high enough for efficient chromatid separation. Perhaps the CTD associates with upstream checkpoint factors that initiate the mechanism of Mad2 activation at NPCs. One further issue that needs to be resolved is that Top2 most likely associates with sites of DNA replication termination (TERs) at the time when the checkpoint is activated. TERs are the sites where replication forks collide and are assumed to be the major sites of decatenation activity in preparation for anaphase [66]. Since Top2 protein concentrates at TERs, how can the CTD of Top2-B44 contribute to Mad2 activation when Mad2 resides at NPCs? This question has not been examined yet, but replication termination does occur at the nuclear periphery in mammalian cells, and these peripheral sites co-localize with active Topo IIα [67,68,69]. If yeast TERs reside at the nuclear envelope in mitosis, this would place these genomic sites in the vicinity of NPCs. Coincidentally, the number of TERs is similar to the number of NPCs in the nuclear envelope, so it is not entirely implausible that these structures coincide [66,70]. Indeed, Top2 has been reported to interact with at least one of the nucleoporins in the NPC that is part of a complex needed for Mad2 recruitment to NPCs [71]. Interactions among Top2 and Mad2 at NPCs and TERs may provide valuable insight into the mechanism of Mad2 activation.

## 6. Prospective Questions

One area that needs heightened focus will be to understand the extent to which the SUMOylated Topo IIα CTD serves as a scaffold for the recruitment of mitotic regulators. Topo IIα activity is required for various processes during mitosis, including chromosome condensation, resolution of sister chromatids, and activation of a metaphase checkpoint. How many of these mitotic processes are facilitated by the binding of proteins to the SUMOylated CTD? Additionally, under what circumstances might different factors be recruited? On the other side, mitotic defects induced by Topo II inhibitors, such as ICRF-193, cause increased SUMOylation and perhaps hyper-SUMOylation of the Topo IIα CTD that mediates the binding of novel cellular proteins through SUMO/SIM interactions. In this respect, it is interesting to consider if the upregulation of Topo IIα SUMOylation may, in part, contribute to defects in mitosis. One intriguing example is the case of Ultra-fine DNA bridge (UFB) formation, observed after ICRF-193 treatment [72,73]. The discovery of PICH-positive DNA threads after ICRF-193 treatment suggested that the Topo IIα activity needed for resolving tangled DNA may be critical to prevent massive UFB formation and that PICH may be required for controlling this process. Interestingly, PICH was found to be a binding protein of SUMOylated chromosomal proteins, and the SIMs of PICH are required for resolving chromatin bridges [74,75]. The upregulation of mitotic SUMOylation after ICRF-193 treatment could induce strong interaction with PICH, and that might somehow contribute to the defect in the resolution of sister chromatids in mitosis after ICRF-193 treatment. A comprehensive analysis of SUMOylation-dependent binding proteins of Topo IIα and utilizing non-SUMOylatable mutant Topo IIα will be able to address these questions directly.

Secondly, it will be interesting to examine if the functional differences between Topo IIα and Topo IIβ are to some degree determined by the different consequences of their SUMOylation. The SUMOylation of both isoforms is known to be increased by catalytic Topo II inhibitors and Topo II poisons [76,77,78]. However, Topo IIβ is then rapidly degraded following further ubiquitination and degradation by the proteasome-mediated mechanism [76,77]. This may be mediated by SUMO-targeted ubiquitin ligase (STUbL)-dependent poly-ubiquitination of the SUMOylated form of Topo IIβ. In contrast, Topo IIα does not show a similar instability after ICRF-193 treatment, suggesting that SUMOylated Topo IIα might not be recognized by STUBL as effectively as Topo IIβ. These outcomes could originate from a difference in the primary structures of the Topo IIα and Topo IIβ CTDs. Perhaps Topo IIβ is SUMOylated by SUMO chains and Topo IIα SUMOylation occurs by single SUMO species. Such differences could collaborate with other functional differences between the Topo IIα and Topo IIβ CTD. For example, as described above, the CTDs have different affinities for DNA. Similarly, the Topo IIβ CTD binds to RNA [79], as does the CTD of the single Drosophila Type II topoisomerase [80], and this ability may be different for the human Topo IIα CTD. The instability of SUMOylated Topo IIβ indicates that this CTD may not function as a scaffold for the recruitment of binding factors. However, it remains possible that a pool of SUMOylated Topo IIβ is stable in some nuclear locations. For example, SUMOylation is known to regulate transcription [81]. Topo IIβ in particular has been revealed to be enriched at gene promoter regions and highly expressed genes and have crucial functions in transcription regulation [82,83]. Many genes become either upregulated or downregulated in postmitotic neurons lacking Topo IIβ and premature neuronal death is observed [84]. Given that Topo IIβ is heavily linked with transcription control, it is possible that the SUMOylated Topo IIβ CTD has distinct binding proteins from SUMOylated Topo IIα and that these binding partners are involved in transcription. Such differences may also explain why Topo IIβ cannot substitute in terms of the mitotic functions of Topo IIα. Future studies will need to identify isoform-specific SUMOylated CTD–SIM interactions.

## Abbreviations

Topo IIα	Topoisomerase IIα
Topo IIβ	Topoisomerase IIβ
CTD	C-terminal domain
UFB	Ultra-fine DNA bridge
XEE	Xenopus egg extract
GFP	Green fluorescent protein
ChT	Chromatin Tether
FRAP	Fluorescence recovery after photo-bleaching
H3T3-Phos	Phosphorylated threonine 3 residue of histone H3
CPC	Chromosome Passenger Complex
SAC	Spindle assembly checkpoint
NPC	Nuclear Pore Complex
TER	Site of DNA replication termination
STUbL	SUMO-targeted ubiquitin ligase
